# Annual Report of the Surgeon-General United States Army

**Published:** 1871-01

**Authors:** 


Annual Report of the Surgeon-General of the United States Army: 1870.

This document is a condensed pamphlet of only ten pages.

The receipts of the Department have been $1,946,878.95, and the expenses very much less, leaving a balance unexpended in the Department of $1,607,064.22.

During the year there have been furnished to soldiers, officers and sailors, 171 artificial legs, 112 arms, 6 feet, and 12 pieces of other apparatus.

The average number of the white troops during the past year on sick report was 1,419 or about 5 per cent. of the army, which is a gratifying state of health, considering that much of it has been in the field, watching the Indians. Of these cases, 263 were for wounds and injuries. The total number of deaths was 374, which was twelve per thousand of mean strength, and is equivalent to a little over one per cent. Among the colored troops the mortality was about fifty per cent. greater, being nineteen per thousand of mean strength. The causes of this difference are not stated.

The record department of the office seems to be progressing with its work, wounds, operations, etc., to the extent of about 25,000 cases having been searched out and recorded. For the purposes of illustration, 3,029 photographs have been printed, 106 wood-cuts made, and 425 pages of the great forthcoming Medical and Surgical History of the War printed. The printing of the first volume of this work, which will contain 750 pages, is nearly completed. The whole of the second, or sugical volume, is also prepared.

This work will contain, among other important surgical matter, 29,572 cases of amputation and 4,775 cases of excision. Owing to the later information of the history of these cases obtained through the Pension Bureau, these statistics will be the most complete, as well as the most extensive, ever published on this subject.

The Army Medical Museum has been much increased, and contains 13,502 specimens, among which are 894 skulls and 34 skeletons.

The museum during the year received about 18,000 visitors, some of whom came expressly for the purpose from Europe, and several great foreign works on surgery are copiously illustrated from specimens in the museum.

The Surgeon-General has received the most flattering testimony from distinguished European surgeons of the value of the documents already published.

